# Analysis of deletional hereditary persistence of fetal hemoglobin/δβ‐thalassemia and δ‐globin gene mutations in Southerwestern China

**DOI:** 10.1002/mgg3.706

**Published:** 2019-05-01

**Authors:** Jie Zhang, Yang Yang, Peng Li, Yuanlong Yan, Tao Lv, Tingting Zhao, Xiaohong Zeng, Dongmei Li, Xiaoyan Zhou, Hong Chen, Jie Su, Tonghua Yang, Jing He, Baosheng Zhu

**Affiliations:** ^1^ Department of Obstetrics and Gynecology The First People’s Hospital of Yunnan Province Kunming China; ^2^ Genetic Diagnosis Center, Yunnan Provincial Key Laboratory for Birth Defects and Genetic Diseases The First People’s Hospital of Yunnan Province Kunming China; ^3^ Affiliated Hospital of Kunming University of Science and Technology Kunming China; ^4^ Department of Hematology The First People’s Hospital of Yunnan Province Kunming China

**Keywords:** bioinformatics analysis, capillary electrophoresis, hereditary persistence of fetal hemoglobin, δ‐thalassemia

## Abstract

**Background:**

Deletional hereditary persistence of fetal hemoglobin (HPFH)/δβ‐thalassemia and δ‐thalassemia are rare inherited disorders which may complicate the diagnosis of β‐thalassemia. The aim of this study was to reveal the frequency of these two disorders in Southwestern China.

**Methods:**

A total of 33,596 subjects were enrolled for deletional HPFH/δβ‐thalassemia, and positive individuals with high fetal hemoglobin (Hb F) level were diagnosed by multiplex ligation‐dependent probe amplification (MLPA). A total of 17,834 subjects were analyzed for mutations in the δ‐globin gene. Positive samples with low Hb A_2_ levels were confirmed by δ‐globin gene sequencing. Furthermore, the pathogenicity and construction of a selected δ‐globin mutation were analyzed.

**Results:**

A total of 92 suspected cases with Hb F ≥5.0% were further characterized by MLPA. Eight different deletional HPFH/δβ‐thalassemia were observed at a frequency of 0.024%. In addition, 195 cases suspected to have a δ‐globin gene mutation (Hb A_2_ ≤2.0%) were characterized by molecular analysis. δ‐Globin gene mutation was found at a frequency of 0.49% in Yunnan. The pathogenicity and construction for a selected δ‐globin mutation was predicted.

**Conclusion:**

Screening of these two disorders was analyzed in Southwestern China, which could define the molecular basis of these conditions in this population.

## 1 INTRODUCTION

Thalassemias are autosomal recessive disorders that result from reduced or absent synthesis of one or more of the hemoglobin chains. Thalassemia can be classified into α‐, β‐, δ‐thalassemia according to the type of globin involved. The human β‐globin gene cluster is arranged in the order as 5′‐ε‐^G^γ‐^A^γ‐δ‐β‐3′. Fetal hemoglobin (Hb F) is a minor hemoglobin that is composed of two α‐ and two γ‐globin chains (α2γ2). Hereditary persistence of fetal hemoglobin (HPFH, OMIM #141749) is caused by mutations in the promoter of the γ‐globin gene (nondeletional HPFH) (Amato et al., 2014) or large deletions in the β‐globin gene cluster (deletional HPFH) (Bilgen, Altiok Clark, Ozturk, Yesilipek, & Keser, 2016). Deletional HPFH and δβ‐thalassemia (deletional HPFH/δβ‐thalassemia) is a rare inherited condition that is characterized by increased Hb F, which results from deletions in the upstream silencer region of the γ‐globin genes or upregulation of the γ‐globin genes (Sankaran, Xu, & Orkin, 2010), as there is no competition from the expression of β‐ and δ‐globin genes. Deletional HPFH/δβ‐thalassemia may lead to the clinical phenotypes of heterogeneous β‐thalassemia with microcytic hypochromic red cell parameters (Pandey et al., 2018).

δ‐Thalassemia is a form of thalassemia with no clinical consequences, and is caused by mutations in the δ‐globin gene that result in lower Hb A_2_ levels (Phylipsen, Gallivan, Arkesteijn, Harteveld, & Giordano, 2011). Although δ‐thalassemia has no clinical significance, the disrupted function of δ‐globin may lead to aberrant measurements of Hb A_2_, and may complicate the diagnosis of β‐thalassemia when relying on the Hb A_2_ measurements of the patient (Amirian et al., 2011). β‐Thalassemia is highly prevalent in Yunnan, China, and has a high genetic heterogeneity (Zhang et al., 2015). Identification of the clinical and hematological phenotypes of these causative mutations and their relative frequency in the population can improve our understanding of broader patterns across human populations. Furthermore, great interest has been paid in methods for predicting protein pathogenicity and structure based on amino acid sequence, which have become more accessible through advancements in bioinformatics tools (Touma et al., 2016). Pathogenic and structural analysis of rare mutations can be predicted using various computational methods (Gomes et al., 2019). For an accurate diagnosis of a rare mutation, it is essential to know how the mutation may affect the function of the gene to result in pathogenic effects through a combination of sequence and structure‐based algorithms.

Both deletional HPFH/δβ‐thalassemia and δ‐thalassemia mutations are related to different ethnic backgrounds (Shang et al., 2017; Xiong et al., 2010). And both deletional HPFH/δβ thalassemia and δ‐globin gene mutations will complicate routine β‐thalassemia screening (Usually, the electrophoretic testing for β‐thalassemia trait was carried out using the following selection criteria: Hb A_2_ ≥ 3.5% and/or Hb F ≥2.0%). As summarized in Globin Gene Server home page (http://globin.cse.psu.edu/hbvar/menu.html), more than 50 deletional HPFH/δβ‐thalassemias and 130 mutations in the δ‐globin gene have been reported to date. However, few reports on deletional HPFH/δβ‐thalassemia and mutations in the δ‐globin gene in Chinese population have been investigated at the molecular level. The aim of this study was to determine the frequency of deletional HPFH/δβ‐thalassemia mutations and δ‐globin gene mutations in Yunnan population. Furthermore, we characterized a rare δ‐globin gene mutation using a comprehensive clinical and structure‐function analysis. These findings are important for accurate thalassemia prenatal diagnosis, as well as for providing molecular insights into new mutations in δ‐globin gene.

## 2 METHODS

### 2.1 Ethical compliance

The study protocol was approved by the medical ethics committee of the First People's Hospital of Yunnan province, and the forms were in accordance with the Declaration of Helsinki. And all patients gave their written informed consent for examination and genetic analyses.

### 2.2 Screening for deletional HPFH/δβ‐thalassemia and δ‐thalassemia

The samples were obtained from individuals who sought genetic counseling or prenatal diagnosis in the First People's Hospital of Yunnan province. Hemoglobin analysis was performed using capillary electrophoresis (CE; Sebia, Paris, France). Internal quality control of the hemoglobin analysis was performed using the control materials provided by the manufacturer. A total of 33,596 subjects (8,139 men and 25,457 women, 6 months to 49 years of age) were screened for deletional HPFH/δβ‐thalassemia using capillary electrophoresis from July 2014 to September 2016. Individuals showing Hb F ≥5.0% were considered to be carriers of deletional HPFH/δβ‐thalassemia (He et al., 2018). A total of 17,834 subjects (4,779 men and 13,055 women, 2–49 years of age) were screened for δ‐thalassemia using capillary electrophoresis from July 2014 to November 2015. The testing for δ‐thalassemia traits was carried out using the following selection criteria: (a) Hb A_2_ levels at or below 2.0% (Hb A_2_ ≤ 2.0%) and (b) low levels of Hb A_2_ associated with a visible second Hb A_2_ fraction (Hassan, Harteveld, Bakker, & Giordano, 2014).

### 2.3 Molecular analyses for deletional HPFH/δβ‐thalassemia

The copy number variation in the β‐globin gene cluster (NC_000011.10) was performed using multiplex ligation‐dependent probe amplification (MLPA) following the manufacturer's instructions (MRC Holland, Amsterdam, the Netherlands). Two commonest deletional HPFH, Chinese ^G^γ(^A^γδβ)^0^ thalassemia and Southeast Asia HPFH (SEA‐HPFH) deletion were identified by Gap‐PCR (He et al., 2018). One intractable case was further sequenced using targeted next‐generation sequencing following methods from a previous report (Shang et al., 2017). Complete blood counts were performed using an automated cell counter (Sysmex, Tokyo, Japan), and whether individuals carried 17 common β‐globin gene mutations were tested using previously described methods (Zhang et al., 2012).

### 2.4 Molecular analyses for δ‐thalassemia

Two fragments of the δ‐globin gene (NG_000007.3) were amplified using the following primers: δ1‐F 5′CTGAGTCAAGACACACATGACAG3′, δ1‐R 5′ TGGTATGCATAATTTGAGTTGTTG3′; δ2‐F 5′ AATATCCTGTCTTTCTCTCCCAAC3′, δ2‐R 5′ TAATTTCTGCTCTTTGGAGGTAG3′ (Amirian et al., 2010). Six of the following known α‐thalassemia deletions were analyzed as previously described (Zhang et al., 2012): ‐α^3.7^ (NC_000016.9:g.223300_227103del), ‐α^4.2^ (NC_000016.9:g.219817_(223755_224074)del), ‐‐^SEA^ (NC_000016.9:g.215400_234700del), α^CS^α (Hb Constant Spring, HBA2:c.427T** > **C), α^WS^α (Hb Weastmead, HBA2:c.369C** > **G), and α^QS^α (Hb Quong Sze, HBA2:c.377T** > **C).

### 2.5 Bioinformatics analysis of rare δ‐thalassemia

We further analyzed the function and structure of the HBD:c.198G > T mutated protein. The isoelectric points (pI) were determined for the normal and mutant δ‐globin variant (monomer) using the Isoelectric Point Calculator (IPC) (http://isoelectric.ovh.org/) (Kozlowski, 2016). The mutated amino acid residue was examined for evolutionary conservation across nine randomly selected species. The pathogenicity of the δ‐globin variant was evaluated using the HumDiv‐trained model in Polyphen‐2 (http://genetics.bwh.harvard.edu/pph2/) and SIFT (http://sift.jcvi.org) (Ernst et al., 2018). The 3D structure of the native δ‐globin protein was modeled using SWISS‐MODEL and PyMol (http://www.pymol.org) (Moriarty et al., 2018; Zhao et al., 2017).

## 3 RESULTS

### 3.1 Screening and molecular analyses of deletional HPFH/δβ‐thalassemia

Among 33,596 samples screened using capillary electrophoresis for deletional (HPFH)/δβ‐thalassemia, 165 positive samples were selected (Hb F ≥5.0%). Eight cases of deletional HPFH/δβ‐thalassemia were found by MLPA (Figure S1 and Figure S2), with a gene frequency of 0.024% (8/33,596). The hematological and electrophoretic characterizations of the eight cases are shown in Table 1.

**Table 1:** The hematological and electrophoretic characterization of eight deletional HPFH/δβ‐thalassemia cases

Case	Age	Sex	Type	MCV (fl)	MCH (pg)	Hb (g/L)	Hb A	Hb A_2_	Hb F
1	38	Male	SEA‐HPFH	70.9	24.9	161	76.7	4.40	18.9
2	2	Male	β^0^ deletion	61.2	20.3	108	71.2	5.10	23.7
3	28	Female	(δβ)^0^ deletion	79.0	27.1	114	69.1	1.90	29.0
4	28	Female	(δβ)^0^ deletion	73.6	25.8	126	70.1	2.00	27.9
5	37	Female	^G^γ(^A^γδβ)^0^ deletion	71.0	23.4	119	82.2	2.40	15.4
6	28	Male	Chinese ^G^γ(^A^γδβ)^0^ deletion	70.0	23.8	153	81.2	2.70	16.1
7	27	Female	Chinese ^G^γ(^A^γδβ)^0^ deletion/ IVS‐I−1 (G > T)	84.2	27.4	66	73.9	2.20	23.9
8	6 months	Female	(εγδβ)^0^ deletion	54.6	17.9	88	90.0	2.70	7.30

Case 1 was identified as a SEA‐HPFH deletion by MLPA and Gap‐PCR. In case 2, screening by MLPA revealed a heterozygous deletion that removed the entire β‐globin gene (β^0^ deletion). Two unrelated individuals (case 3 and case 4) showed a similar (δβ)^0^ **‐**deletion. A ^G^γ(^A^γδβ)^0^ **‐**deletion was found in case 5. Two unrelated individuals (cases 6 and case 7) had a ^G^γ(^A^γδβ)^0^ deletion, and this was confirmed as Chinese ^G^γ(^A^γδβ)^0^ thalassemia by Gap‐PCR. Furthermore, case 7 was a compound heterozygous for Chinese ^G^γ(^A^γδβ)^0^ and IVS‐I‐1 (G > T) (HBB:c.92 + 1G>T). Case 8 showed a “discontinuous” (εγδβ)^0^ deletion, where the size of the deletion ranged from OR51M1‐1 probe to hemoglobin subunit beta (*HBB*)‐up probe; however, among this deletion region, the hemoglobin subunit gamma (*HBG)*2–3 and *HBG*1‐up probes detected a fragment. Therefore, this deletion was considered to be a “discontinuous” (εγδβ)^0^ deletion, and its sequence was further confirmed by targeted next‐generation sequencing (Figure S2).

### 3.2 Screening and molecular analyses for δ‐thalassemia

Out of a total of 17,834 samples screened using capillary electrophoresis, 195 samples had low Hb A_2_ (Hb A_2_ ≤2.0%) or a second Hb A_2_ fraction, and were selected for further molecular diagnosis. Seven types of δ‐globin mutations were found in 87 δ‐thalassemia patients (Figure S3). δ‐Globin mutations were not detected in the remaining 108 subjects. The frequency of δ‐thalassemia was 0.49% (87/17,834) in the population of Yunnan (Table 2). The most frequent genotype was −77 (T > C) (HBD:c.‐127T > C), which was found in 77 δ‐thalassemia carriers (88.51%, 77/87). Other mutations linked to δ‐thalassemia in the Yunnan population were as follows: −30 (T > C) (HBD:c.‐80T > C), Initiation codon Met > Ile (HBD:c.3G > A), HBD:c.127T > C, HBD:c.394C > G, HBD:c.198G > T, and HBD:c.347C > T. We previously reported HBD:c.394C > G and HBD:c.198G > T in another study as novel mutations.

**Table 2:** The frequency and Hb variant types of δ‐thalassemia

Mutation	HGVS nomenclature	Hb A (%)	Hb A2 (%)	Variant Hb A2 (%)	Type	Frequency
−77 (T > C)	HBD:c.−127T > C	97.7 ± 0.99	1.42 ± 0.09	–	δ0	88.5%, 77/87
−30 (T > C)	HBD:c.−80T > C	97.7 ± 1.14	1.63 ± 0.32	–	δ+	3.45%, 3/87
Initiation codon Met > Ile	HBD:c.3G > A	98.5 ± 0.0	1.20 ± 0.0	–	δ0	2.30%, 2/87
CD 42 (T > C), Hb A2‐Huadu	HBD:c.127T > C	98.5 ± 0.14	1.50 ± 0.14	–	δ0 or δ+	2.30%, 2/87
CD 65 (G > T), Hb A2‐Yunnan	HBD:c.198G > T	98.0	1.30	0.70	δ+	1.15%, 1/87
CD 115 (C > T)	HBD:c.347C > T	98.6	1.4	–	–	1.15%, 1/87
CD 131 (C > G), Hb A2‐Puer	HBD:c.394C > G	97.3	1.30	1.40	–	1.15%, 1/87

The number of subjects for each Hb A_2_ value, genotypes, and the relationship between the two parameters are indicated in Table 2. Individuals carrying the HBD:c.394C > G and HBD:c.198G > T mutations had a variant Hb A_2_ band. All samples carrying the −77 (T > C) mutation had Hb A_2_ values below 1.6% (Hb A_2_ ≤ 1.6%). One sample was a compound heterozygote for −77 (T > C) and ‐α^3.7^ with Hb A_2_ value of 1.4% (other electrophoretic characterization: Hb A 97.7%, Hb F 0.9%).

### 3.3 Bioinformatics analysis results

The HBD:c.198G > T (p.Lys65Asn) mutation was selected for further bioinformatics analysis. The proband was a 31‐year‐old Chinese woman. Her hematological characteristics were as follows: RBC 4.5×10^12^/L, Hb 13.8g/dl, MCV 91.0fl, MCH 30.3pg, RDW‐CV 13.1%, and MCHC 333g/L. The predicted pIs of the wild type and mutant type were 7.42 and 6.87 respectively (Figure S4a). The Lys residue in CD 65 (K65) was conserved across diverse species, except the elephant (Figure S4b). Polyphen‐2 predicted that the potential pathogenicity of HBD:c.198G > T was benign (Figure S4c). However, SIFT analysis predicted this mutation to be deleterious (Figure S4d).

**Figure 1:**
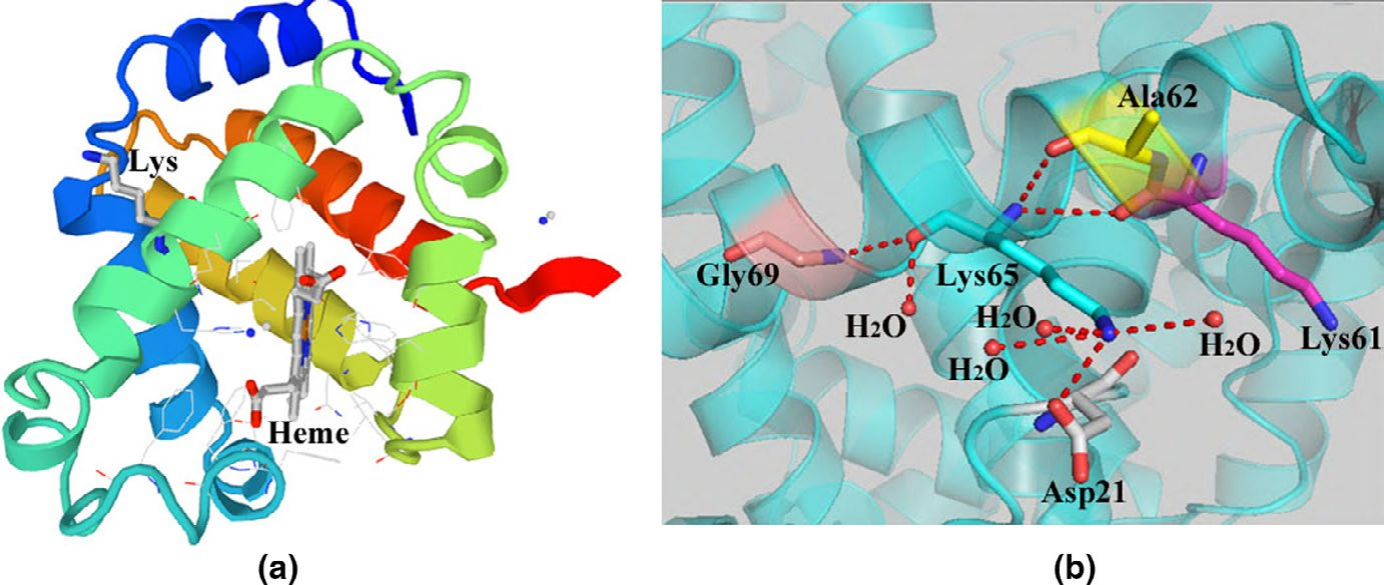
The 3D models of HBD:c.198G > T evaluated by SWISS‐MODEL and PyMol. (a) SWISS‐MODEL prediction of δ‐globin protein structure. Heme: protoporphyrin IX containing FE. (b) The structural environment of K65. The wild type structure of K65 (blue) and intermonomer contacts of four residues (D21, K61, A62 and G69). There was no evidence showing the involvement of K65 in any vital interactive network

SWISS‐MODEL predicted that the Lys residue was predicted to be located on the α‐helix (Figure 1a). The 3D structure predicted by PyMol found that K65 was in contact with four amino acid residues [Asp21 (D21), Lys61 (K61), Ala62 (A62), and Gly69 (G69)] and 4 H_2_O atoms (Figure 1b). None of the bioinformatics assays suggested that K65 was involved in an interactive network.

## 4 DISCUSSION AND CONCLUSIONS

In the areas where thalassemia is prevalent, detecting deletional HPFH/δβ‐thalassemia and δ‐globin gene mutation is important, as coexistence of HPFH or δ‐thalassemia with α‐ or β‐thalassemia will lead to the misdiagnosis and missed diagnosis of thalassemia (Chen, Huang, Zhou, & Li, 2017). Deletional HPFH/δβ‐thalassemia and δ‐thalassemia mutations are variable within different human populations (Pandey et al., 2018). Characterizing the spectrum and frequency of deletional HPFH/δβ‐thalassemia and δ‐thalassemia is vital for prenatal diagnosis programs for thalassemia. Here, we characterized deletional HPFH/δβ‐thalassemia and mutations in the δ‐globin gene in a Southwestern Chinese population. Subsequently, a series of bioinformatics methods was used to predict the pathogenicity and structure of a rare δ‐globin gene mutation that was identified in the population. The combined molecular approach in this report enabled a guideline for genetic counseling and prenatal diagnosis to be developed. To our knowledge, this is the first report on deletional HPFH/δβ‐thalassemia and δ‐thalassemia associated with the hematological parameters in Southwestern China.

Individuals with deletional HPFH/δβ‐thalassemia have elevated Hb F levels (Patel, Dehury, Purohit, Meher, & Das, 2015). In this study, 165 subjects with Hb F level ≥5.0% (1.15%, 165/33,596) were screened positively by capillary electrophoresis, and eight cases of deletional HPFH/δβ‐thalassemia were found by MLPA. For the undiagnosed 157 positive samples, we predicted the following: (a) some positive samples may have been the result of a nondeletional point mutation in the γ‐globin genes (Wienert et al., 2017); (b) some regulatory factors such as Krüppel‐like factor 1 (*KLF1*) and B cell CLL/lymphoma 11A (*BCL11A*) can modulate fetal‐to‐adult globin switching. Mutations in *KLF1* can result in significantly boosted Hb F levels in normal individuals (Gallienne, Dréau, Schuh, Old, & Henderson, 2012). And some polymorphisms in *BCL11A* can lead to higher Hb F levels (Bauer et al., 2013); and (c) a high selection criteria such as Hb F ≥10.0% may be adopted to avoid false positives for deletional HPFH/δβ‐thalassemia, as suggested by a previous report (Mayuranathan et al., 2014). In our study, all confirmed deletional HPFH/δβ‐thalassemia samples had Hb F values higher than 15.0%.

The frequency obtained in study (0.024%, 8/33,596) should be regarded as the frequency of deletional HPFH/δβ‐thalassemia but not β‐globin cluster deletions. Some β‐globin cluster deletions, such as the 118 kb Filipino deletion with a normal Hb F 1.7% cannot be screened by capillary electrophoresis (Cui, Azimi, Baysdorfer, Vichinsky, & Hoppe, 2013). The frequency of deletional HPFH/δβ‐thalassemia (0.024%) was lower than other regions in China, such as Guangxi (0.21%) (He et al., 2018). Among the eight samples, one case of SEA deletion, one case of β^0 ^deletion, two cases of (δβ)^0^ **‐**deletion, one case of ^G^γ(^A^γδβ)^0^ **‐**deletion, two cases of Chinese ^G^γ(^A^γδβ)^0^ deletion, and one case of (εγδβ)^0^ **‐**deletion were identified. All eight samples had typical β‐thalassemia phenotypes with hypochromic and microcytic erythrocytes due to deletions in the β‐globin gene or promoter. Case 7 was a compound heterozygote for the Chinese ^G^γ(^A^γδβ)^0^ deletion and IVS‐I‐1 (G > T). As a result, this patient had typical β‐thalassemia intermedia symptoms with increased Hb F levels (Table 1). Case 8 had an intractable (εγδβ)^0^ **‐**deletion, and targeted next‐generation sequencing was used to confirm the “discontinuous” deletion fragment (Figure S2).

The widespread use of capillary electrophoresis in routine laboratory test to separate and quantify Hb fractions facilitated the diagnosis of δ‐thalassemia (Villegas et al., 2017). The frequency of δ‐thalassemia in the Yunnan population is 0.49%, which is higher than other populations in Southern China (0.4%, 152/40,863) (Liu et al., 2013). δ‐Globin gene mutation was found in 87 cases (44.62%, 87/195). The remaining cases did not have mutations in the δ‐globin gene (55.38%, 108/195), and their anomalous Hb A_2_ levels may have resulted from integration artifacts or to other causes such as iron‐deficiency anemia and *KLF1* mutation (El‐Agouza, Abu Shahla, & Sirdah, 2002; D. Liu et al., 2014). The most frequent δ‐globin gene mutation was −77 T > C (88.51%, 77/ 87), followed by −30 T > C (3.45%, 3/87) and Initiation codon Met > Ile (2.30%, 2/87). This result was similar to the previously reported in the population of China (Liu et al., 2013), but different from that reported in other ethnic populations in Europe. In the United Kingdom population, the most prevalent δ‐globin gene mutations were HBD:c.49G > C, HBD: c.82G > T, and HBD:c.410G > A (Khalil et al., 2014).

Based on bioinformatics and structural analysis, the pathogenicity and construction of a rare δ‐globin gene mutation were analyzed. Hb A_2_ is a minor adult hemoglobin composed of two α and two δ protein chains (α2δ2). The individuals who have δ‐globin gene mutations do not usually have clinical phenotypes because of the physiologically lower expression levels of the δ‐globin gene (Alayi, Van Dorsselaer, Epting, Bisse, & Schaeffer‐Reiss, 2014). The AAG > AAT mutation at CD 65 of the δ‐globin gene leads to a missense mutation (Lys > Asn). This amino acid change results in a predicted pI change from 7.42 to 6.87, and this would result in the individual carrying the mutation to have a second Hb A_2_ fraction with a different pI. K65 is conserved among the species that were investigated (Figure S4b), indicating a potentially important biological function of this residue. Pathogenicity analysis showed discrepant results: SIFT predicted the mutation to be deleterious, while Polyphen‐2 predicted it to be benign. These results suggested that the pathogenicity prediction of δ‐globin gene mutation should not be completely based on these two software models.

The 3D models evaluated by SWISS‐MODEL and PyMol showed that the K65 was located in a helix near the periphery of the protein. Based on the results from SWISS‐MODEL and PyMol, we observed that K65 was not positioned in a complex or important interaction network. Therefore, the substitution of the Lys residue should not significantly disrupt the structure and function of Hb A_2_. In case of the β‐globin gene, the counterpart of Hb A_2_‐Yunnan is the Hb J Sicilia [beta 65 (E9) Lys > Asn, HBB:c.198G > T]. A previous study also had shown that those heterozygous for Hb J Sicilia had no clinically significant problems (Ricco et al., 1974). The molecular structure of Hb A is similar to that of Hb A_2_ with a slight subunit modification (Sen et al., 2004). Taking all the results together, there is ample support for the idea that missense variant should not disrupt Hb A_2_ structure obviously and p.K65N mutation in δ‐globin gene should be benign.

In conclusion, this is the first report on the frequency and spectrum of deletional HPFH/δβ‐thalassemia and mutations in the δ‐globin gene in a Southwestern Chinese population. Bioinformatic analysis of a rare mutation characterized the potential changes at the protein‐level. Our study will provide a guideline for genetic counseling and prenatal diagnosis.

## CONFLICT OF INTEREST

The author reports no conflict of interest in this work.
