# Supplementary material for: Analysis of deletional hereditary persistence of fetal hemoglobin/δβ‐thalassemia and δ‐globin gene mutations in Southerwestern China
Source: Mol Genet Genomic Med. 2019 May 1;7(6):e706. doi: 10.1002/mgg3.706 (PMC6565566; doi:10.1002/mgg3.706)
Supplement: Supplementary file 1 [file MGG3-7-e706-s001.pdf]

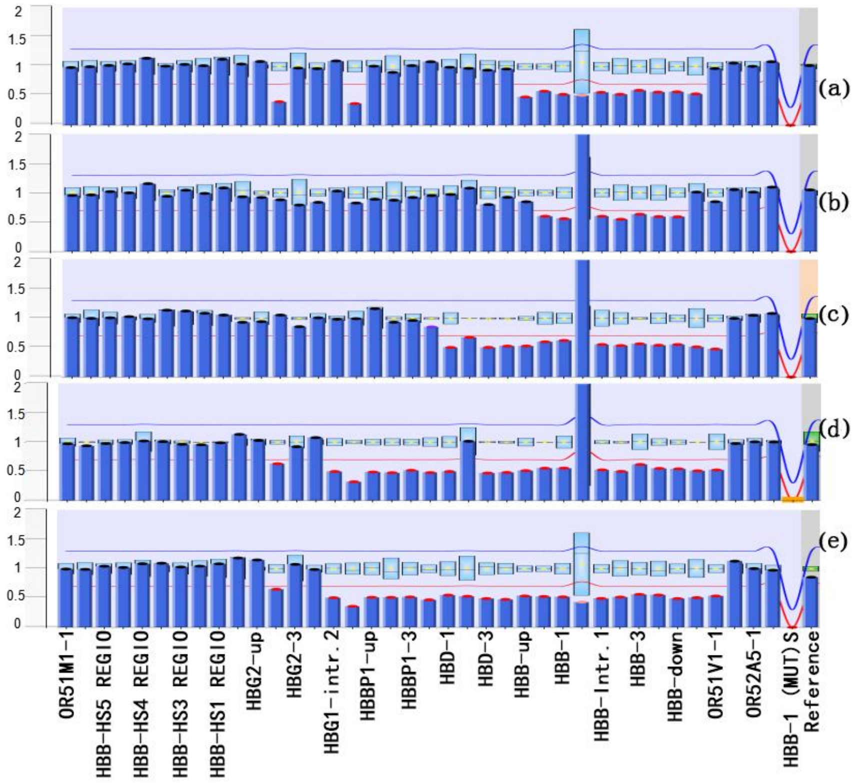

FIGURE S1 MLPA analysis of the β-globin cluster (NC_000011.10). The peak height ratio for deletion fragment and normal fragment were set at 0.5 and 1.0, respectively. (a) Case 1, SEA-HPFH. (b) Case 2, β^0 deletion. (c) Case 3 and case 4, $(\delta\beta)^0$ deletion. (d) Case 5, ${}^G\gamma({}^A\gamma\delta\beta)^0$ deletion. (e) Case 6 and case 7, Chinese ${}^G\gamma({}^A\gamma\delta\beta)^0$ deletion.

Zhang et al. Figure S1
