# Supplementary material for: Analysis of deletional hereditary persistence of fetal hemoglobin/δβ‐thalassemia and δ‐globin gene mutations in Southerwestern China
Source: Mol Genet Genomic Med. 2019 May 1;7(6):e706. doi: 10.1002/mgg3.706 (PMC6565566; doi:10.1002/mgg3.706)
Supplement: Supplementary file 2 [file MGG3-7-e706-s002.pdf]

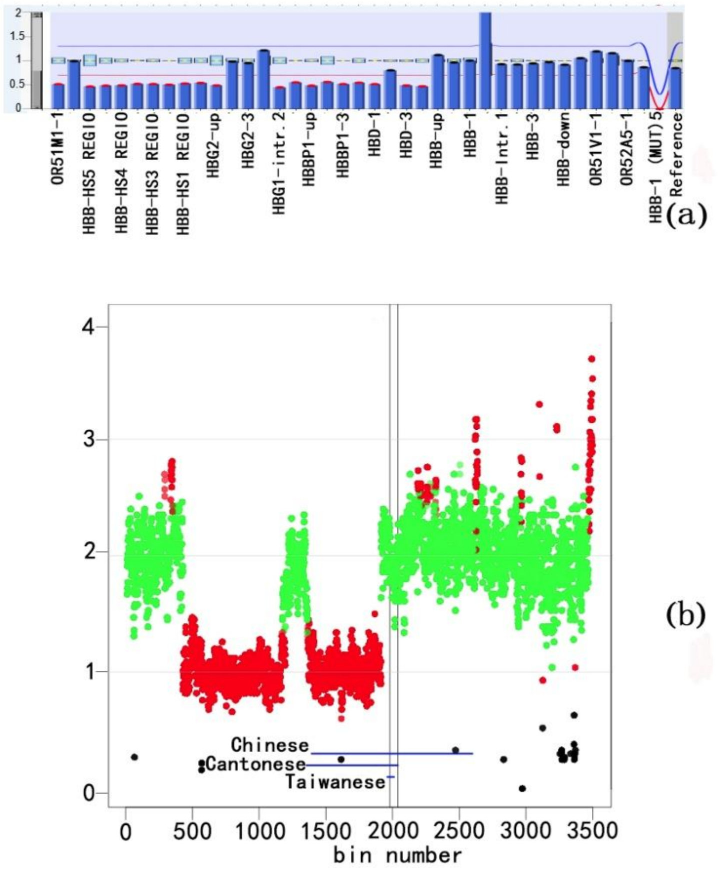

FIGURE S2 The (εγδβ)^0 deletion confirmed by MLPA and targeted next-generation sequencing. (a) (εγδβ)^0 deletion (case 8) showing a heterozygous deletion between probe OR51M1-1 to *HBB*-up, and presence of fragments from *HBG*2-3 to *HBG*1-up probe among this deletion region. The peak height ratio for deletion fragment and normal fragment were set at 0.5 and 1.0, respectively. (b) Next-generation sequencing validation of the "discontinuous" (εγδβ) ^{0} deletion. The peak height ratio for the deletion fragment and normal fragment were set at 1.0 and 2.0, respectively. Bin was defined as window, which were 30bp in length and shared 5bp overlaps between nearby ones. Bin number 500 equaled to 15000bp (30bp × 500).
