# Supplementary material for: Analysis of deletional hereditary persistence of fetal hemoglobin/δβ‐thalassemia and δ‐globin gene mutations in Southerwestern China
Source: Mol Genet Genomic Med. 2019 May 1;7(6):e706. doi: 10.1002/mgg3.706 (PMC6565566; doi:10.1002/mgg3.706)
Supplement: Supplementary file 3 [file MGG3-7-e706-s003.pdf]

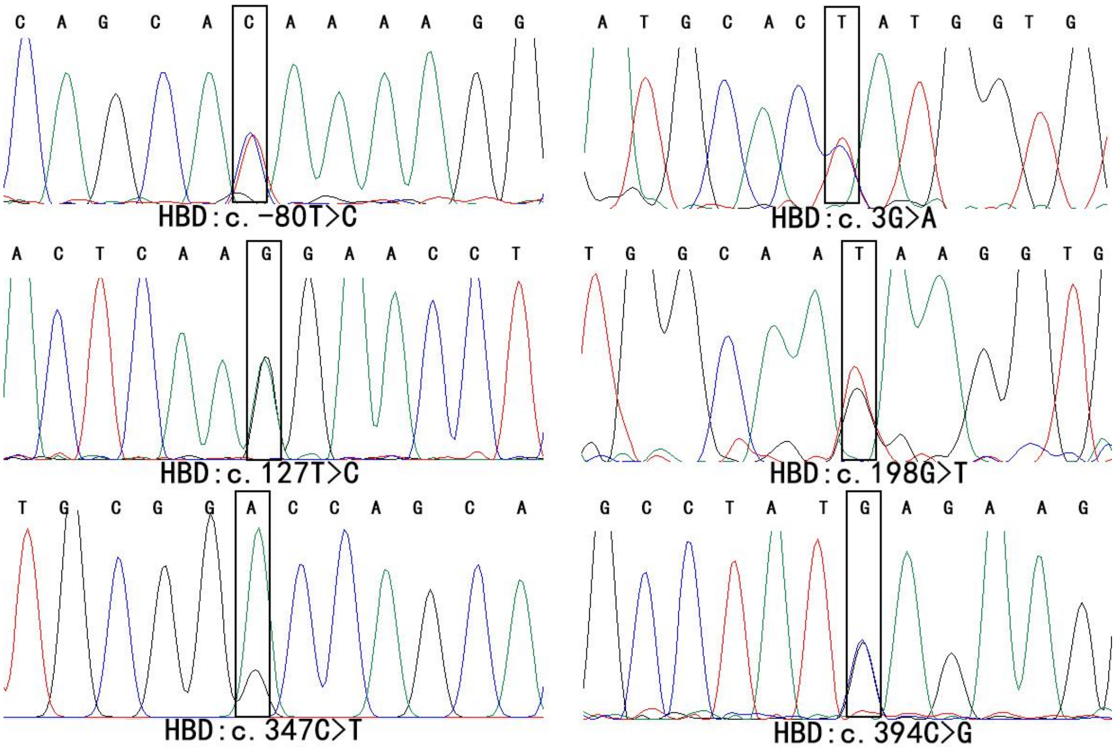

FIGURE S3 DNA sequencing profiles of the δ-mutations (NG_000007.3) analyzed. HBD:c.-80T>C, HBD:c.127T>C, and HBD:c.347C>T were reverse sequencing; HBD:c.3G>A, HBD:c.198G>T, and HBD:c.394C>G were forward sequencing.

Zhang et al. Figure S3
