# Supplementary material for: Analysis of deletional hereditary persistence of fetal hemoglobin/δβ‐thalassemia and δ‐globin gene mutations in Southerwestern China
Source: Mol Genet Genomic Med. 2019 May 1;7(6):e706. doi: 10.1002/mgg3.706 (PMC6565566; doi:10.1002/mgg3.706)
Supplement: Supplementary file 4 [file MGG3-7-e706-s004.pdf]

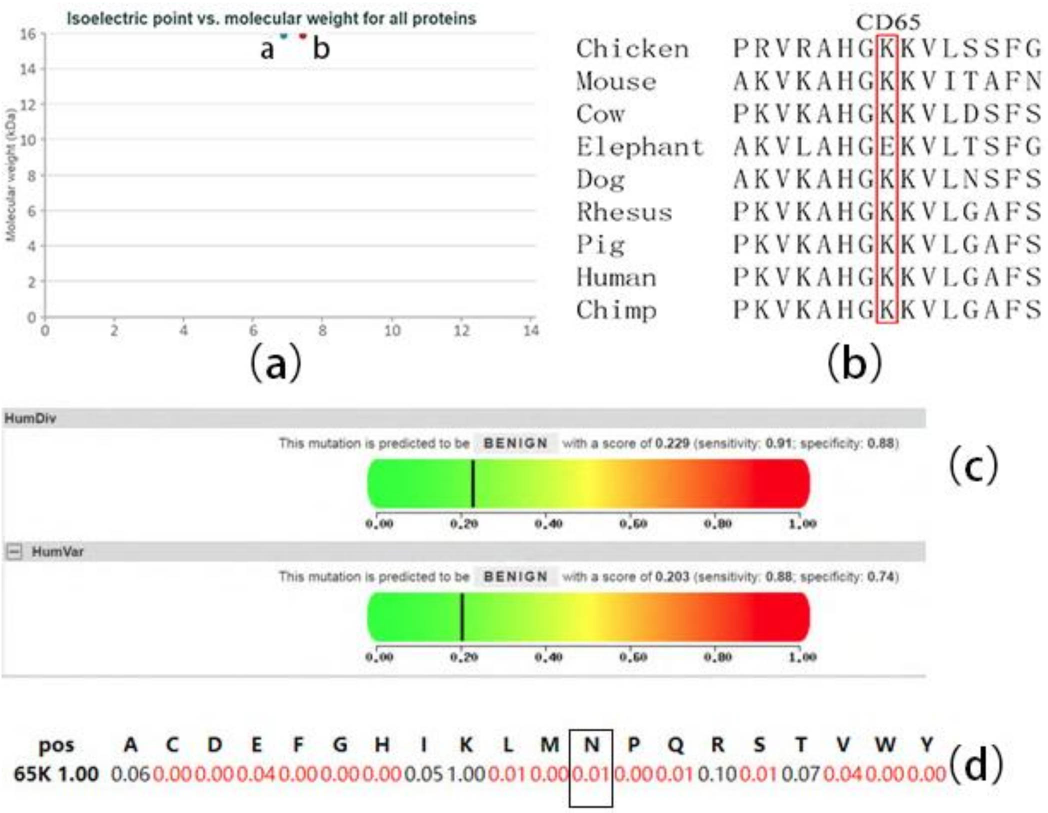

FIGURE S4 Bioinformatics analysis of HBD:c.198G>T. (a) The pI of mutant Hb A_2 (a, 6.87) compared to normal Hb A_2 (b, 7.42). (b) Conservation of K65 amino acid sequence among vertebrates. In all of the nine species, the elephant had a non-conserved Lys residue at the site. (c) The SIFT prediction analysis of the HBD:c.198G>T with a score of 0.229, suggesting that the rare mutation is benign. The HumDiv model was preferred as recommended by software (score from 0.00-1.00, with lower score indicating pathogenicity). (d) The Polyphen-2 prediction analysis of HBD:c.198G>T scored 0.01, suggesting that the mutation is pathogenic. For PolyPhen-2, lower scores indicate that the mutation is benign.
